# Presenting complaints and patient volume in an Emergency Department during one year

**DOI:** 10.1186/1757-7241-20-S2-P41

**Published:** 2012-04-16

**Authors:** Rasmus Carter-Storch, Ulrik Frydkjær-Olsen, Christian Backer-Mogensen

**Affiliations:** 1 Emergency department, Kolding Hospital, Denmark

## Background

Emergency departments (EDs) have a different case-mix than traditional medical and surgical departments. Knowledge of what complaints patients present with at EDs and their incidence is needed. Previous studies on ED case-mix have largely focused on the incidence of different diagnoses. This study aims to create a number of categories that cover the majority of presenting complaints and to establish their volume.

## Methods

A retrospective cohort study of all admissions to Kolding ED during one year. The department covers acute medical, surgical, orthopaedic and vascular surgical admissions to the hospital. The data was retrieved from the electronic system Cetrea, where nurses wrote what complaints or tentative diagnoses the patients were presented with by the referring doctor. The authors developed a list of symptoms, and sorted the patients accordingly. The authors each sorted a share of the patients individually, but met several times to adjust the list and unify the sorting. The patients were each assigned to a maximum of two presenting complaints and/or two tentative diagnoses.

## Results

During 2010 10,070 patients were admitted at Kolding ED. 40.5 % of admissions were medical, 26.8 % were surgical, 14.1 % were orthopaedic and 4.0 % were vascular surgical. The patients presented with a total of 11,220 complaints and tentative diagnoses, 74.7 % presented with one or more complaints and 43.5 % presented with one or more tentative diagnoses. These were initially placed in 77 groups of complaints and 44 tentative diagnoses. These groups were aggregated into 31 larger categories comprising 92.6 % of the total number of complaints and tentative diagnoses. The remaining 7.4 % included among others postoperative complications, procedures, various pains and missing information.

## Conclusion

It is possible to create 31 categories that cover the majority of presenting complaints in an ED. If one knew what diagnoses the patients in each category were discharged with, it would possible to create specific “packages” for each category, helping the health personnel to choose e.g. which laboratory tests to order, in order to cover most discharge diagnoses. This is a topic of further studies.

